# A Method for Direct Measurement of the First-Order Mass Moments of Human Body Segments

**DOI:** 10.3390/s101009155

**Published:** 2010-10-12

**Authors:** Yusaku Fujii, Kazuhito Shimada, Koichi Maru, Junichi Ozawa, Rong-Sheng Lu

**Affiliations:** 1 Department of Electronic Engineering, Gunma University, 1-5-1 Tenjin-cho, Kiryu, Gunma, 376-8515, Japan; 2 Space Medical Operations, Japan Aerospace Exploration Agency (JAXA) Tsukuba Space Center, Tsukuba, 305-8505, Japan; 3 School of Instrument Science and Opto-electronic Engineering, Hefei University of Technology, Hefei, Anhui, 230009, China

**Keywords:** body segment inertial parameter, center of mass, biomechanics

## Abstract

We propose a simple and direct method for measuring the first-order mass moment of a human body segment. With the proposed method, the first-order mass moment of the body segment can be directly measured by using only one precision scale and one digital camera. In the dummy mass experiment, the relative standard uncertainty of a single set of measurements of the first-order mass moment is estimated to be 1.7%. The measured value will be useful as a reference for evaluating the uncertainty of the body segment inertial parameters (BSPs) estimated using an indirect method.

## 1. Introduction

The knowledge of body segment inertial parameters (BSPs) is required in the field of biomechanics. These parameters can be accurately estimated using dual x-ray absorptiometry (DXA) [1–3]. Although some DXA-based two-dimensional computation methods can be considered a direct measurement, the instrumentation for DXA is expensive and is not available to most researchers. In addition, the DXA-based three-dimensional BSP computation [4] is not a real direct measurement since it is necessary to assume mass densities to reconstruct a three-dimensional mass distribution. On the other hand, many simple models for estimating BSPs have also been proposed [5,6], and data derived from DXA are sometimes used as the standard.

In this paper, a simple and direct method for measuring the first-order mass moment, M^(1)^ = ML, is proposed, where M is the mass of a body segment and L is the length from the axis of rotation to the position of the center of mass (CoM) of the segment.

In the proposed method, the first-order mass moment of a human body segment M^(1)^ is obtained from the two positions of the CoM of the entire body, in which the body segment under test has two different rotation angles. Only one precision scale and one digital camera are used in the experiment.

The first-order mass moment obtained with the proposed method can be used to evaluate the validity of the values of the BSPs obtained from other non-direct measurement methods. The moment value is also used for the posture error analysis in measuring human body masses in spacecraft with Space Scale [7–9].

## 2. Methods

**Figure 1.**
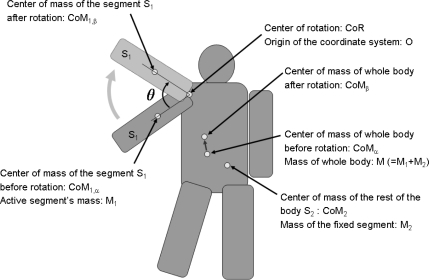
Principle of the method (top-down view).

Figure 1 shows the principle of the top-down view of the proposed method. The center of mass of the entire body can be expressed using the center of mass of the body segment S_1_ and that of the rest of the body S_2_ as follows:

(1) $$\text{CoM M}={\text{CoM}}_{1}M_{1}+{\text{CoM}}_{2}M_{2}$$

where CoM, CoM_1_, and CoM_2_ are the center of mass of the entire body, of the body segment S_1_, and of the rest of the body S_2_, respectively. M, M_1_, and M_2_ are the mass of the entire body, of the body segment S_1_, and of the rest of the body S_2_, respectively.

Equation (1) can be expressed as follows when the body segment under test S_1_ has two different rotation angles:

(2) $${\text{CoM}}_{\alpha }M={\text{CoM}}_{1\alpha }M_{1}+{\text{CoM}}_{2}M_{2}$$

(3) $${\text{CoM}}_{\beta }M={\text{CoM}}_{1,\beta }M_{1}+{\text{CoM}}_{2}M_{2}$$

where suffixes α and β correspond to the two different states of the body obtained when the segment under test S_1_ has two different rotation angles. When the rotation occurs in the horizontal plane, CoM_1,β_ can be expressed using the rotation matrix R(θ) and CoM_1,α_ as follows:

(4) $${\text{CoM}}_{1,\beta }=(R(\theta ))({\text{CoM}}_{1,\alpha })$$

(5) $$R(\theta )=\left(\begin{array}{cc} \text{cos}\theta & -\text{sin}\theta \\ \text{sin}\theta & \text{cos}\theta \end{array}\right)$$

where θ is the rotation angle of the segment under test S_1_ from the initial state (expressed using suffix α) to the final state (expressed using suffix β). On the basis of Equations (2) and (3), CoM_2_ M_2_ can be eliminated as follows:

(6) $$({\text{CoM}}_{\alpha }-{\text{CoM}}_{\beta })M=({\text{CoM}}_{1,\alpha }-{\text{CoM}}_{1,\beta })M_{1}$$

(7) $$({\text{CoM}}_{\alpha }-{\text{CoM}}_{\beta })M=(I-R(\theta ))({\text{CoM}}_{1,\alpha })M$$

where I is the identity matrix. Then, the first-order mass moment M^(1)^_1_ of the segment under test S_1_ can be calculated using the rotation matrix R(θ)^−1^; the center of mass of the entire body before and after the rotation of segment S_1_, *i.e.*, CoM_α_ and CoM_β_; and the mass of the entire body M as follows:

(8) $$M_{1}^{\left(1\right)}=|{\text{CoM}}_{1,\alpha }|M_{1}=|{\left(I-R(\theta )\right)}^{-1}({\text{CoM}}_{\alpha }-{\text{CoM}}_{\beta })|\;M$$

**Figure 2.**
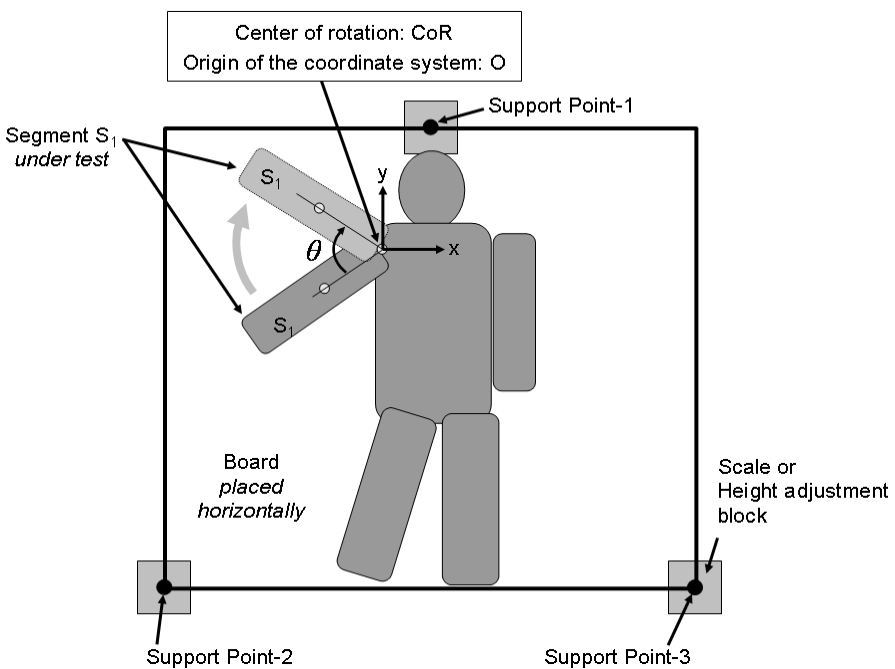
Setup for measuring the center of mass CoM of a subject body.

The values of CoM at the two different states are measured using a common technique [10,11]. Figure 2 shows the setup for measuring the CoM of an entire body.

The center of rotation of segment S_1_, CoR, is set as the origin of the coordinate system, O. The entire body is placed on a horizontal board, which is supported by the following three points: Support Point-1, Support Point-2, and Support Point-3. The weights applied at these three points are measured using a precision scale (capacity 3100 g, resolution: 0.1 g, precision: 1.5 g, model: GX-30KR, manufacturer: A and D Co., Ltd. (Japan)) one by one, while the attitude of the body is fixed. During the measurement, the three support points are supported by the scale or the height adjustment blocks whose total height is equal to that of the scale. CoM is calculated as follows:

(9) $$\text{CoM}=(P_{P1}M_{P1}+P_{P2}M_{P2}+P_{P3}M_{P3})/(M_{P1}+M_{P2}+M_{P3})$$

where P_P1_, P_P2_, and P_P3_ are the horizontal positions of Support Point-1, Support Point-2, and Support Point-3, respectively. M_P1_, M_P2_, and M_P3_ are the readings of the scale at Support Point-1, Support Point-2, and Support Point-3, respectively. The horizontal positions P_P1_, P_P2_, and P_P3_; the center of rotation CoR (O); and the angle of rotation θ are manually measured using the pictures taken by the digital network cameras [image capture resolution: 640 × 480, model: BL-C1, manufacturer: Panasonic Co., Ltd. (Japan)] placed at a distance of approximately 2.4 meters above the board. The rotation angle is manually measured using the printed overlapped images that are taken by the fixed digital camera with the help of a straightedge and protractor. Here, we did not compensate the distortion of the image due to the perspective projection of the camera when we estimate the center of rotation. The distortion is thought to be negligible because the camera is placed sufficiently far from the board (approximately 2.4 m, compared to the body thickness of less than 0.2 m and lens center line to margin length of about 0.5 m).

## 3. Experiment with a Dummy Mass

### 3.1. Experiments

**Figure 3.**
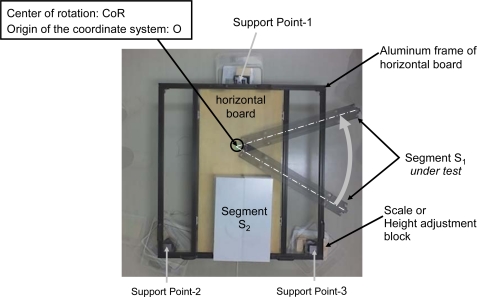
Overlapped pictures used in the analysis (subject: dummy mass, M_1_: 3.013 kg, M^(1)^_1_: 116.3 kg·cm).

The first-order mass moment M^(1)^ of a dummy mass is evaluated for demonstrating the performance of the proposed method. Figure 3 shows the overlapped pictures, which correspond to a set of measurements.

An aluminum bar (mass: 3.013 kg, length: 77.2 cm) is used as the segment under test S_1_, which simulates an arm or a leg under the test. The other aluminum block with a mass of 52.066 kg is also used as segment S_2_, which simulates the rest of the human body. Both S1 and S2 are placed on the horizontal board with a mass of 11.627 kg. The center of rotation CoR and the origin of the coordinate system O are defined at the end of segment S_1_. The first-order mass moment M^(1)^ of segment S_1_ around the origin O (the center of rotation CoR) is calculated to be 116.3 kg·cm. In the experiment, 30 sets of measurements are conducted with a rotation angle between 30° and 180°.

### 3.2. Results

**Figure 4.**
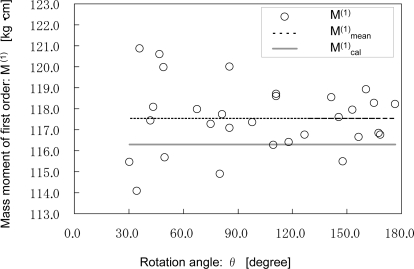
Relationship between the 30 measured values of M^(1)^, their mean value M^(1)^_mean_, and the calibrated value M^(1)^_cal_ *versus* the rotation angle θ.

Figure 4 shows the relation between the 30 measured values of M^(1)^, their mean value M^(1)^_mean_, and the calibrated value M^(1)^_cal_ *versus* the rotation angle. The mean value M^(1)^_mean_ and the calibrated value M^(1)^_cal_ are 117.6 kg·cm and 116.3 kg·cm, respectively. The standard deviation of the measured values of M^(1)^ is 1.619 kg·cm, which corresponds to 1.4% of the calibrated value M^(1)^_cal_. The root mean square value (RMS value) of the difference between the measured values of M^(1)^ and the calibrated value M^(1)^_cal_ is 2.0 kg·cm, which corresponds to 1.7% of the calibrated value M^(1)^_cal_. This value is considered to be the relative standard uncertainty of a single set of measurements of M^(1)^.

## 4. Experiments with a Human Body

### 4.1. Experiments

**Figure 5.**
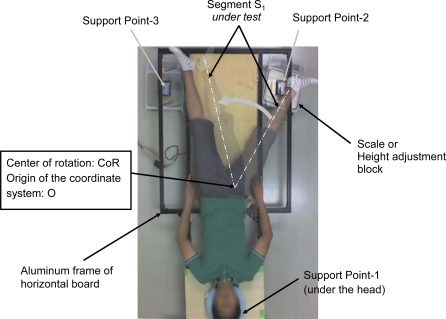
Overlapped pictures used in the analysis.

The first-order mass moment M^(1)^ of a leg of a human body is measured using the proposed method. Figure 5 shows the overlapped pictures, which correspond to a set of measurements. In the experiments, 15 sets of measurements are conducted with a constant rotation angle of 48.8°. The total mass of the subject human body, including the clothes, and that of the frame are 63.460 kg and 15.569 kg, respectively. The rotation center RoC is estimated from the overlapped pictures such as Figure 5.

### 4.2. Results

**Figure 6.**
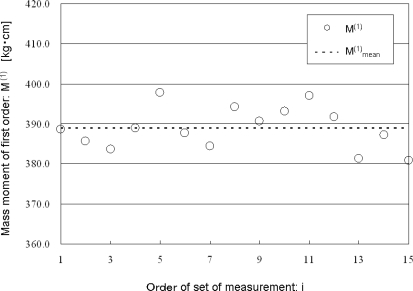
Relationship between the 15 measured values of M^(1)^ and their mean value M^(1)^_mean_ *versus* the order of the set of measurements i.

Figure 6 shows the relationship between the 15 measured values of M^(1)^ and their mean value M^(1)^_mean_ *versus* the order of the set of the measurement. The mean value of the 15 measured values of M^(1)^ is 388.8 kg·cm. The standard deviation of the 15 measured values of M^(1)^ is 5.3 kg·cm, which corresponds to 1.4% of the mean value.

## 5. Discussion

The relative standard uncertainty of a single set of measurements of M^(1)^ in the dummy mass experiment is estimated to be 1.7%. The standard deviations of the values of M^(1)^ in the experiments using the dummy mass for 30 measurements with various rotation angles and the human body for 15 measurements with a constant rotation angle are both 1.4% of the mean value. Although these error values are not directly comparable, the random errors of both the experiments are thought to be at the same level. This indicates that the change of posture of the other part of the human body during one set of measurements can be ignored even when the leg is rotated. With regard to the bias error, the bias error in the human body experiment might be slightly larger than that in the dummy mass experiment since the accuracy of the estimation of the rotation center CoR in the human body experiment might be lesser than that in the dummy mass experiment. In the experiments, the rotation canter CoR and the rotation angle θ are manually measured using the printed overlapped images that are taken by the fixed digital camera with the help of a straightedge and protractor. The accuracy of estimating the rotation center will be improved with more careful consideration [12,13], such as considering 3-dimensional anatomical structure.

The measured value will be useful as a reference for evaluating the uncertainty of the body segment inertial parameters (BSPs) estimated using an indirect method, in which the mass and the center of mass of a segment are estimated [5, 6]. In the evaluation, the value of the first-order mass moment M^(1)^ of a body segment, which is directly measured using the proposed method, is compared with the value, which is calculated from the mass, the center of mass and the center of rotation estimated using the indirect method.

In the experiment, only one scale is used for measuring the weights applied at the three supporting points of the board. Therefore, during the measurements of the three points, the subject should not be moved; this requires a certain amount of effort in the case of the human subject. If three scales are used, then the weights at the three points can be obtained immediately and the measurement time can be reduced.

## 6. Conclusions

A simple and direct method for measuring the first-order mass moment M^(1)^ of the human body segment has been proposed. By using the proposed method, the first-order mass moment of the body segment can be directly measured by using only one precision scale and one digital camera. The relative standard uncertainty of a single set of measurements of M^(1)^ in the dummy mass experiment is estimated to be 1.7%. The measured value will be useful as a reference for evaluating the uncertainty of the body segment inertial parameters (BSPs) estimated using an indirect method.
